# Vasoactive Intestinal Peptide in Early Spondyloarthritis: Low Serum Levels as a Potential Biomarker for Disease Severity

**DOI:** 10.1007/s12031-015-0517-6

**Published:** 2015-02-25

**Authors:** Iria V. Seoane, Eva Tomero, Carmen Martínez, Rosario Garcia-Vicuña, Yasmina Juarranz, Amalia Lamana, Elena Ocón, Ana M. Ortiz, Nieves Gómez-León, Isidoro González-Álvaro, Rosa P. Gomariz

**Affiliations:** 1 Departamento de Biología Celular, Facultad de Biología, Universidad Complutense de Madrid, Antonio Novais, no. 2, Ciudad Universitaria, 28040 Madrid, Spain; 2 Servicio de Reumatología, Hospital Universitario de la Princesa, Instituto de Investigación Sanitaria la Princesa, Madrid, Spain; 3 Departamento de Biología Celular, Facultad de Medicina, Universidad Complutense de Madrid, Madrid, Spain; 4 Servicio de Radiodiagnóstico, Hospital Universitario de la Princesa, Instituto de Investigación Sanitaria la Princesa, Madrid, Spain

**Keywords:** Neuroimmunomodulation, Spondyloarthritis, Vasoactive intestinal peptide, Prognosis biomarker, Bath Ankylosing Spondylitis Disease Activity Index

## Abstract

Spondyloarthritis (SpA) is a family of inflammatory diseases sharing clinical, genetic, and radiological features. While crucial for tailoring early interventions, validated prognostic biomarkers are scarce in SpA. We analyze the correlation between serum levels of vasoactive intestinal peptide (VIP) and disease activity/severity in patients with early chronic inflammatory back pain. The study population comprised 54 patients enrolled in our early chronic inflammatory back pain register. We collected demographic information, clinical data, laboratory data, and imaging findings. VIP levels were measured by enzyme immunoassay in serum samples from 162 visits. The association between independent variables and VIP levels was analyzed using longitudinal multivariate analysis nested by patient and visit. No significant differences were observed in VIP levels between these two groups. Lower levels of VIP were significantly associated with a higher Bath Ankylosing Spondylitis Disease Activity Index (BASFI) score, presence of bone edema in magnetic resonance imaging (MRI) scan, and lower hemoglobin levels. Coexistence of cutaneous psoriasis was independently associated with lower VIP levels, and similar trend was observed for enthesitis. We conclude that SpA patients with low serum VIP levels had worse 2-year disease outcome, suggesting that serum VIP levels could be a valid prognostic biomarker.

## Introduction

Spondyloarthritis (SpA) is a family of interrelated rheumatic diseases that share clinical and radiological manifestations; the most prevalent and paradigmatic member of the group is ankylosing spondylitis (Dougados and Baeten 2011). SpA comprises a wide variety of skeletal and extra-skeletal phenotypes that are associated with the presence of the HLA-B27 antigen, which accounts for familial association and can explain recent hallmark findings on pathogenesis (Colbert et al. 2010). Compared with joint involvement in rheumatoid arthritis (RA), inflammation in SpA mainly takes the form of enthesitis and commonly results in bone formation and ankylosis rather than bone erosion (Marzo-Ortega et al. 2002; McGonagle et al. 2014). Nevertheless, the consequences of these changes also lead to functional limitations, poor quality of life, loss of working hours, and even decreased life expectancy (Braun and Pincus 2002; Fabreguet et al. 2012). Prognosis is often poorer because of the delay in diagnosis, especially in patients with isolated axial involvement (Rudwaleit et al. 2005). This was particularly true until 5–10 years ago, when diagnosis could only be confirmed based on radiological damage (van der Linden et al. 1984). New classification criteria enable SpA to be diagnosed before radiologic evidence of disease is available (Rudwaleit et al. 2009b), and magnetic resonance imaging (MRI) now plays a key role in the early identification of patients with SpA (Bennett et al. 2009). Nonetheless, diagnosis of non-radiologic SpA remains a challenge, since, in most cases, the first manifestation is low back pain, a common complaint in the general population (Carmona et al. 2001; Deyo and Weinstein 2001).

Biologics, mainly TNF blockers, are the second major advance in the management of SpA (Machado et al. 2013). However, given the considerable heterogeneity in the clinical manifestations of these disorders, ranging from self-limiting to chronic severe and progressive forms (Dougados and Baeten 2011), and concerns over safety and cost, the use of TNF blockers should be restricted to patients with an optimal risk-benefit ratio. Prognostic markers such as C-reactive protein (CRP) levels, smoking, HLA-B27 positivity, and structural damage at diagnosis have been associated with progression of radiological damage (Chung et al. 2012; Marzo-Ortega et al. 2009; Poddubnyy et al. 2012); however, validated biomarkers for predicting progression are lacking and early detection of candidates for TNF blockade remains difficult.

Vasoactive intestinal peptide (VIP) is a molecule of the neuroendocrine-immune network with demonstrated immunoregulatory properties (Gomariz et al. 2006; Leceta et al. 2007). Treatment with VIP has been shown to reduce the severity of arthritis in murine models (Delgado et al. 2001; Juarranz et al. 2005) and improves the course of other inflammatory models in mice (Abad et al. 2003; Jimeno et al. 2010; Li et al. 2006; Lodde et al. 2006). In human assays, in vitro use of VIP regulated differentiation of T cells, modulated the balance of proinflammatory and anti-inflammatory cytokines (Gutierrez-Canas et al. 2008), and decreased expression of several proinflammatory pathways in synovial fibroblasts (Carrion et al. 2011, 2014; Gutierrez-Canas et al. 2006). We recently reported that early arthritis patients with low baseline serum VIP levels have a more severe disease course and require more intensive treatment with disease-modifying anti-rheumatic drugs (DMARDs) (Martinez et al. 2014). In addition, reduced VIP expression has been observed in the synovial fluid of osteoarthritis patients with a poorer disease course (Jiang et al. 2012). The aim of this study, therefore, was to elucidate the potential role of VIP serum levels as a biomarker of severity in early inflammatory back pain.

## Material and Methods

### Ethics Statement

The register protocol for the early chronic inflammatory back pain (CIBP) clinic at Hospital Universitario La Princesa was reviewed and approved by the Ethics Committee for Clinical Research at Instituto de Investigación Sanitaria La Princesa. Consecutive patients who attended the early CIBP clinic from June 2010 to June 2013 were recruited. All patients were informed about the study and signed an informed consent form before their inclusion in the register.

### Patients

The study sample comprised 54 patients from the register. The inclusion criteria are inflammatory back pain (IBP) for more than 3 months and less than 2 years, and symptom onset before age 45 years.

The register protocol comprised three prospective visits (baseline, 1 and 2 years). The data recorded at baseline include gender, age, ethnicity, and educational and occupational status. At each visit, we systematically collect clinical and laboratory data, as follows: date of onset of IBP, specific features of SpA (enthesitis, arthritis, uveitis, psoriasis, and inflammatory bowel disease), previous infections and family history of SpA. Patients were asked to complete the Bath Ankylosing Spondylitis Disease Activity Index (BASDAI) (Garrett et al. 1994) and Bath Ankylosing Spondylitis Functional Index (BASFI) (Calin et al. 1994). The laboratory parameters recorded include HLA-B27 positivity, CRP levels, erythrocyte sedimentation rate (ESR), alkaline phosphatase (ALP), and hemoglobin (Hb) among other parameters. In addition, serum samples were taken and stored at −80 °C. Radiographs of the sacroiliac joints and the cervical and lumbar spine (lateral view) were taken at every visit. Radiological sacroiliitis was defined according to the modified New York criteria (van der Linden et al. 1984). MRI of the sacroiliac joints is performed at baseline to assess the presence of the active inflammatory lesions (bone marrow edema) according to the Assessment of SpondyloArthritis international Society (ASAS)/Outcome Measures in Rheumatology (OMERACT) definition (Rudwaleit et al. 2009a).

Information related to the treatment established for each patient (DMARDs, nonsteroidal anti-inflammatory drugs [NSAIDs], biologic agents, and/or corticosteroids) is also recorded. The data were entered into an electronic database.

Of the 54 patients initially enrolled, 37 were diagnosed with SpA based on the rheumatologist’s clinical judgment, 34 fulfilled the ASAS criteria for SpA (Rudwaleit et al. 2009b) and 13 the New York criteria for ankylosing spondylitis (van der Linden et al. 1984). After 2 years of follow-up, the 17 patients who did not satisfy the criteria for a diagnosis of SpA were considered to have non-specific chronic low back pain (NLBP).

## Measurement of Serum VIP Level

VIP levels were assessed using a commercially available competitive enzyme-linked immunosorbent assay (ELISA) kit (Phoenix Pharmaceuticals, Karlsruhe, Germany). Briefly, the serum samples were freeze-dried and dissolved in ELISA buffer (2:1), added to an immunoplate coated with a secondary antibody, and incubated with biotinylated VIP and a primary antibody whose Fab fragment was competitively bound by both biotinylated peptide and targeted peptide in samples. After washes, we added streptavidin-horseradish peroxidase to each well. During this incubation, the enzyme catalyzed the oxidation of the substrate solution. We terminated the enzymatic reaction using a stop solution and measured absorbance at 450 nm. A standard curve of known concentration was established. The concentration of VIP in the samples was determined by extrapolation to this standard curve and application of the corresponding dilution factor. Samples from each patient were assayed twice. The minimum detectable concentration was 0.12 ng/ml, with an intra-assay and interassay variation of ≤5 and 15 %, respectively. We previously reported that there was no significant correlation between VIP serum level and frozen time (Martinez et al. 2014).

### Statistical Analysis

Normally distributed quantitative variables were represented as the mean ± standard deviation (SD), while non-normally distributed variables were represented as the median and interquartile range (IQR). Variables with a normal distribution were analyzed using the *t* test; variables with a non-normal distribution were analyzed using the Mann-Whitney test or Kruskal-Wallis test. Categorical variables were expressed as percentages and significance levels between groups were established using the χ^2^ test or the Fisher exact test.

In order to better determine the association between heterogeneity in serum VIP levels and clinical variables, we fitted a population-averaged model by means of generalized linear models nested by patient and visit using the * xtgee* command of Stata 12 for Windows (StataCorp LP, College Station, TX, USA). Since the raw variable serum VIP level did not show a Gaussian distribution, data were normalized through logarithmic transformation. Variables displaying a *p* value <0.15 in the bivariate analysis were included in the multivariate analysis. Age and sex were included because previous studies had shown that these variables can act as confounders (Martinez et al. 2014). In addition, considering the interassay variability of ELISA, the model was also adjusted for the variations in assay plate VIP levels. Thereby, the final models were constructed using the quasi-likelihood under the independence model information criterion and the Wald test (Pan 2001) after removing all variables with a *p* value above 0.15.

## Results

### Characteristics of Patients with Early SpA

The study population comprised 54 patients with either SpA (*n* = 37) or NLBP (*n* = 17). Table 1 shows the characteristics of the patients at baseline. The main differences between the two groups were that those diagnosed with SpA more often had a family history of SpA, HLA-B27 positivity, inflammation, structural damage in the sacroiliac MRI scan or radiographs, higher PCR levels, and lower levels of hemoglobin than patients with NLBP. However, no significant differences between these groups were detected for the functional indices, disease activity, or other laboratory variables (Table 1).

**Table 1:** Baseline characteristics of patients with spondyloarthritis or chronic low back pain

	Spondyloarthritis (*n* = 37)	Low back pain (*n* = 17)	Total (*n* = 54)	*p* value
Age* (years)	35.8 ± 10.7	40.8 ± 9.7	37.5 ± 10.6	0.09
Gender** (female)	25 (67.6)	8 (47.1)	33 (61)	NS
Ethnicity** (Caucasian)	26 (70.3)	12 (70.6)	38 (70.4)	NS
Family history of SpA**	14 (37.8)	1 (5.9)	15 (27.8)	0.015
HLA-B27 (+)**	21 (56.7)	3 (17.7)	24 (44.4)	0.007
Enthesitis**	16 (43.2)	3 (17.7)	19 (35.2)	0.067
Arthritis**	11 (29.7)	1 (5.9)	12 (22.2)	0.05
Uveitis**	3 (8.1)	1 (5.9)	4 (7.4)	NS
Psoriasis**	4 (10.8)	1 (5.9)	5 (9.3)	NS
IBD **	2 (5.4)	0 (0.0)	2 (3.7)	NS
Previous infections**	3 (8.1)	0 (0.0)	3 (5.6)	NS
Sacroiliitis MRI**	22 (59.5)	0 (0.0)	22 (40.7)	<0.001
Sacroiliitis X-ray**	12 (33.3)	0 (0.0)	12 (22.2)	NS
BASDAI*	40.5 ± 23.1	36.4 ± 22.9	39.4 ± 22.7	NS
BASFI***	29 [12–45]	25.5 [11–50]	26 [12–45]	NS
CRP (mg/dl) *	0.99 ± 2.3	0.2 ± 0.25	0.7 ± 0.2	0.048
ESR (mm/h)*	23.3 ± 18.8	15.6 ± 13.0	21.0 ± 17.5	0.082
ALP (U/l)*	62.8 ± 19.4	67.2 ± 18.2	64.2 ± 18.9	NS
Hb (g/dl)*	13.2 ± 2.4	14.6 ± 1.3	13.6 ± 2.2	0.023
VIP (pg/ml)***	250 [209 – 280]	292 [230–337]	251 [212–303]	NS

Data are shown as the mean ± standard deviation (SD), frequency *n* (%), or median and [interquartile range]

*IBD* inflammatory bowel disease, *MRI* magnetic resonance imaging, *BASDAI* Bath Ankylosing Spondylitis Disease Activity Index, *BASFI* Bath Ankylosing Spondylitis Functional Index, *CRP* C-reactive protein, *ESR* erythrocyte sedimentation rate, *ALP* alkaline phosphatase, *Hb* hemoglobin, *VIP* vasoactive intestinal peptide, *NS* non-significant

Statistical significance was established by * Student’s *t* test, **χ^2^ or ***Kruskal-Wallis test for a *p* value <0.05

No significant differences were detected in serum VIP levels between SpA patients and NLBP patients at baseline (*p* = 0.36) (Table 1). In addition, no relevant variation in VIP levels was observed during follow-up in either patient subset. We observed a non-significant trend toward increased VIP levels with age (data not shown), as we had reported previously in RA patients (Martinez et al. 2014). However, we did not observed significant differences by gender (data not shown), opposite to what we had observed in RA patients (Martinez et al. 2014).

### Low Serum VIP Levels are Associated with Different Features of SpA

**Fig. 1:**
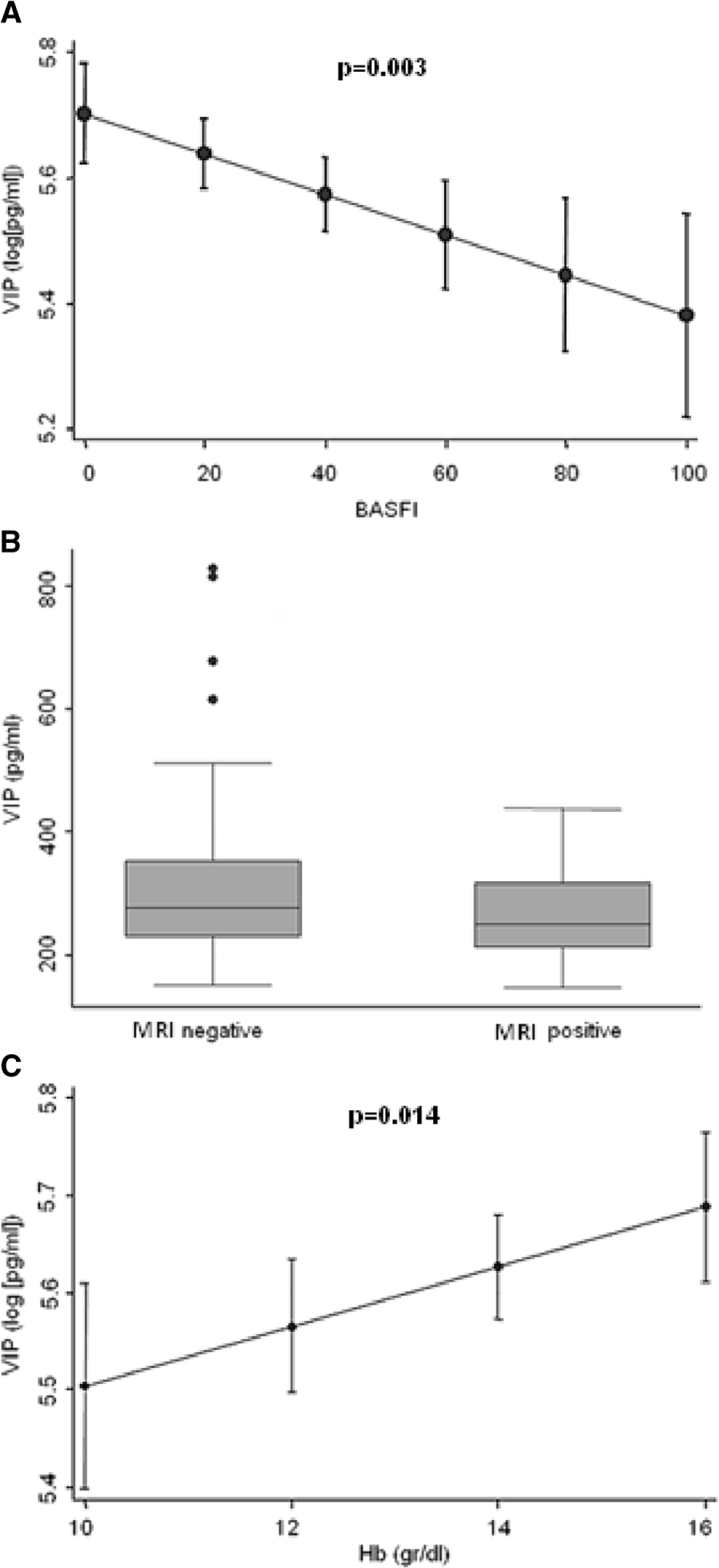
Correlation between VIP serum levels during follow-up and clinical, radiological, and analytical variables. **a** Correlation between serum VIP levels during follow-up and the BASFI score. Data are shown as the mean value of VIP (normalized by logarithmic transformation) for each BASFI value adjusted for the other variables included in the multivariate analysis (*solid dot*). The figure also shows the 95 % confidence interval (*lines above and below the dot*). **b** Association between VIP levels and radiological variables. Distribution of VIP serum levels of the study cohort based on the presence or absence of lesions associated with spondylitis on MRI. Data are represented as the interquartile range (p75 *upper edge of the box*, p25 *lower edge*, p50 *midline*), as well as the p90 (*line above the box*) and p10 (*line below the box*) of the serum VIP levels. *Dots* represent outliers. This figure illustrates the association between VIP serum levels and MRI findings shown at Table 2 where statistical significance is reported. **c** Association between VIP levels and hemoglobin levels. Data are shown as the mean value of VIP (normalized by logarithmic transformation) for each value of hemoglobin (Hb) adjusted for the other variables included in the multivariate analysis (*solid dot*) and the 95 % confidence interval (*lines above and below the dot*). **a**, **b** The values were estimated using the *margins* command of Stata 12, following a multivariate analysis for longitudinal data nested by visit and patient using the *xtgee* command

The BASDAI and BASFI scores were associated with serum VIP levels in the bivariate analysis, although only BASFI maintained this association in the multivariate analysis (Table 2). Patients with lower VIP levels had significantly higher BASFI scores (Fig. 1a), which indicate more severe disability. In addition, patients with inflammation on the MRI scan also had lower serum levels of VIP (Fig. 1b). This association was significant and independent in the multivariate analysis (Table 2). Furthermore, hemoglobin, which is associated with disease activity, was also significantly associated with serum VIP levels (Table 2 and Fig. 1c).

**Table 2:** Multivariate analysis of variables associated with VIP serum levels

	β Coeff. ± SE	CI (95 %)	*p* value
Enthesitis	−0.104 ± 0.058	−0.217 to 0.010	0.075
Uveitis	0.383 ± 0.106	0.176 to 0.591	<0.001
Psoriasis	−0.227 ± 0.094	−0.411 to −0.044	0.015
Sacroiliitis MRI	−0.150 ± 0.057	−0.262 to −0.038	0.009
Biological therapy	0.184 ± 0.118	−0.048 to 0.417	0.120
BASFI	−0.003 ± 0.001	−0.005 to −0.001	0.003
Hb (g/dl)	0.031 ± 0.012	0.006 to 0.055	0.014

Results of multivariate analysis performed with data from SpA patients and NLBP patients. Only variables with *p* value <0.15 in the respective bivariate analysis were included. Variables with *p* value <0.15, in the respective bivariate analysis, that were not included in the final model of the multivariate analysis are as follows: age, family history of SpA, HLA-B27(+), arthritis, CRP, and ESR

*β Coeff*. β coefficient of Wald test, *SE* standard error, *CI* confidence interval

**Fig. 2:**
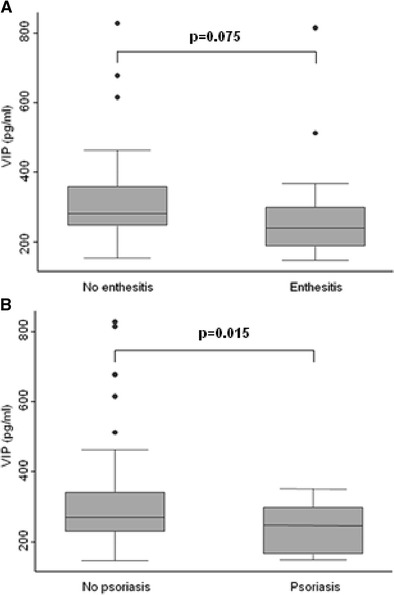
Decreased VIP serum levels are associated with clinical manifestations of enthesitis (**a**) and psoriasis (**b**). The figure illustrates the association between VIP serum levels and these clinical manifestations shown at Table 2 where statistical significance is reported. Data are represented as the interquartile range (p75 *upper edge of the box*, p25 *lower edge*, p50 *midline*), as well as the p90 (*line above the box*) and p10 (*line below the box*) of serum VIP levels. *Dots* represent outliers

The presence of enthesitis or cutaneous psoriasis was also independently associated with lower serum VIP levels (Table 2, Fig. 2).

Only two patients in our study had inflammatory bowel disease; a non-significant trend toward lower levels of VIP was observed in both (data not shown). By contrast, patients who had experienced uveitis displayed significantly higher levels of VIP (Table 2).

In order to determine how robust these results were, we also perform a multivariate analysis restricted to the SpA patients group. Most associations remain similar as those described herein (Table 3).

**Table 3:** Multivariate analysis of variables associated with VIP serum levels in patients diagnosed from spondyloarthritis

	β Coeff. ± SE	CI (95 %)	*p* value
Enthesitis	−0.133 ± 0.057	−0.245 to 0.020	0.020
Uveitis	0.165 ± 0.112	−0.054 to 0.385	0.140
Psoriasis	−0.203 ± 0.094	−0.388 to −0.018	0.032
Sacroiliitis MRI	−0.173 ± 0.058	−0.286 to −0.059	0.003
Biological therapy	0.175 ± 0.123	−0.066 to 0.416	0.150
BASFI	−0.003 ± 0.001	−0.006 to −0.001	0.017
Hb (g/dl)	0.023 ± 0.013	0.003 to 0.049	0.081

Results of multivariate analysis performed with data from SpA patients. Only variables with *p* value <0.15 in the respective bivariate analysis were included. Variables with *p* value <0.15, in the respective bivariate analysis, that were not included in the final model of the multivariate analysis are as follows: age, family history of SpA, HLA-B27(+), arthritis, CRP, and ESR

*β Coeff*. β coefficient of Wald test, *SE* standard error, *CI* confidence interval

Finally, only three patients required TNF blockers during follow-up, and we observed a non-significant trend toward higher levels of VIP after TNF blockade (data not shown). No significant association was observed with the prescription of DMARDs (sulfasalazine and methotrexate).

## Discussion

We provide the first evidence of the association between low serum VIP levels and increased disease severity in patients with early SpA, namely, impaired functional status, bone edema in MRI, and a more intense inflammatory burden (anemia, psoriasis, IBD, and enthesitis). Our results provide additional support to in vitro and animal models by reinforcing the role of VIP as a major regulator of the immune system in autoimmune diseases (Gomariz et al. 2006; Gutierrez-Canas et al. 2008).

Although a previous study had described higher levels of serum VIP in ankylosing spondilitis patients compared to healthy donors (Nalbant et al. 2011), our results showed no differences between SpA and nonspecific low back pain patients. Possible explanations for this divergence are differences between both populations in gender distribution, race, selection of controls, and early versus long-term disease stage. In addition, our findings are consistent with those of our previous observation that patients with early arthritis and low VIP serum levels have a more severe disease course (Martinez et al. 2014). Although variability in the VIP gene has not been reported as a genetic trigger of autoimmune disorders, several findings raise the possibility that once the disease has developed, low expression of VIP could lead to poorer outcome (Jiang et al. 2012; Martinez et al. 2014).

One of the various unmet needs in SpA is the availability of reliable biomarkers both to distinguish inflammatory back pain from the more prevalent mechanical pain and to determine prognosis (Almodovar et al. 2014; de Vlam 2010). New ASAS diagnostic criteria and increased use of MRI have mitigated previous diagnostic limitations, although clinicians still need tools to predict disease activity and progression or to decide which patients would benefit from biologic therapies. In this context, measurement of serum VIP levels could be a useful tool for detecting a subpopulation with a greater functional and inflammatory burden.

Our results raise the question of why BASFI, instead of BASDAI, was associated with low VIP levels in the multivariate analysis. Each index correlated well with the other, especially in early disease, although they have the disadvantage of focusing only on the patient’s perspective (Ward et al. 2013). Bearing in mind that other objective measures of disease activity such as MRI findings or hemoglobin levels were included in the multivariate analysis, it can be assumed that BASDAI did not provide additional information to the statistical model (Kiltz et al. 2012; Machado et al. 2012). In fact, it is well known that functional limitation at diagnosis is associated with a worse prognosis of SpA (Landewe et al. 2009). Therefore, the negative correlation between serum VIP levels and evolution of BASFI during follow-up is an interesting finding, since predicting long-term disability remains a challenge in patients with early SpA.

The association between low serum VIP levels and extra-articular features of SpA, such as psoriasis and enthesitis, further suggests the role of VIP as a biomarker of severity, since both impair quality of life and increase treatment requirements. In addition, VIP levels in the two patients with inflammatory low back pain and IBD were below the median VIP concentration in the whole population, although the difference was not statistically significant. Conversely, we found that the correlation between VIP and uveitis contrasted with the expected trend. Nevertheless, among patients with uveitis, the patient with the highest VIP levels did not fulfill the criteria for SpA. When this patient was excluded, serum VIP levels in patients with uveitis did not exceed the median concentration in the whole population.

Drug therapy for controlling axial inflammation after failure of NSAIDs remains almost restricted to TNF blockers (van der Heijde et al. 2011); the role of DMARDs in severe peripheral forms is limited. Consequently, in recent years, one of the main objectives in the treatment of SpA has been to identify patients with an optimal safety profile and cost-benefit ratio for TNF antagonists. An interesting result in our study was that treatment with anti-TNF agents seemed to increase serum VIP levels. This effect did not reach statistical significance, probably owing to the small number of patients requiring this treatment. However, it is noteworthy that we previously reported the same effect of TNF blockade on serum VIP levels in patients with RA: the increase was statistically significant, probably because of the higher number of patients treated with TNF blockers (Martinez et al. 2014). Several features point to serum VIP levels as a putative marker of the need to prescribe TNF blockers, as follows: (a) high BASFI scores and the presence of enthesitis have been identified as predictors of response to therapy with these drugs (Vastesaeger et al. 2011); (b) anemia is associated with inflammatory activity in SpA, and improvements in hemoglobin levels have been reported after anti-TNF treatment (Furst et al. 2013; Niccoli et al. 2012); (c) low serum VIP levels have been associated with high BASFI scores and the presence of enthesitis and anemia; and (d) administration of TNF blockers increases serum VIP levels. Furthermore, it would be of particular interest to determine whether the increase in serum VIP levels reported with TNF blockers is class-specific or is also observed in autoimmune disorders treated with other biological therapies, such as blockade of interleukin (IL)-6 or IL-12 signaling.

Our study is subject to a series of limitations. First, the sample is small, although the longitudinal design did enable us to evaluate a considerable number of observations and to better control individual variations. Second, we were unable to detect differences in VIP levels between patients with SpA and those with NLBP, even when the first visit was analyzed separately (data not shown). In addition, we have used a mixed population of patients that were finally diagnosed from SpA and others with NLBP. However, when patients with suspicion of SpA are attended for the first time at the clinic this is the challenge and establishing early a prognosis may be helpful despite a definite diagnosis has not been done. Nevertheless, our results were similar when analyzing the whole population or only those patients with SpA diagnosis.

In summary, validated biomarkers of the severity of SpA are scarce and insufficient to properly identify and classify early SpA patients according to their treatment requirements and prognosis. Consequently, new validated biomarkers are needed to achieve these goals. In this framework, the main finding of our study is that patients with early inflammatory low back pain and low serum VIP levels experience a higher inflammatory burden and worse functional outcome during the first 2 years of their disease course.
